# The germ cell-specific RNA binding protein RBM46 is essential for spermatogonial differentiation in mice

**DOI:** 10.1371/journal.pgen.1010416

**Published:** 2022-09-21

**Authors:** Natoya J. Peart, Taylor A. Johnson, Sungkyoung Lee, Matthew J. Sears, Fang Yang, Mathieu Quesnel-Vallières, Huijuan Feng, Yocelyn Recinos, Yoseph Barash, Chaolin Zhang, Brian P. Hermann, P. Jeremy Wang, Christopher B. Geyer, Russ P. Carstens

**Affiliations:** 1 Department of Medicine, Perelman School of Medicine, University of Pennsylvania, Philadelphia, Pennsylvania, United States of America; 2 Department of Anatomy and Cell Biology, Brody School of Medicine, East Carolina University, Greenville, North Carolina, United States of America; 3 Department of Biomedical Sciences, University of Pennsylvania School of Veterinary Medicine, Philadelphia, Pennsylvania, United States of America; 4 Department of Genetics, Perelman School of Medicine, University of Pennsylvania, Philadelphia, Pennsylvania, United States of America; 5 Department of Systems Biology and Department of Biochemistry and Molecular Biophysics, Columbia University, New York, New York, United States of America; 6 Department of Biology, University of Texas at San Antonio, San Antonio, Texas, United States of America; 7 East Carolina Diabetes and Obesity Institute at East Carolina University, Greenville, North Carolina, United States of America; The Lundquist Institute, UNITED STATES

## Abstract

Control over gene expression is exerted, in multiple stages of spermatogenesis, at the post-transcriptional level by RNA binding proteins (RBPs). We identify here an essential role in mammalian spermatogenesis and male fertility for ‘RNA binding protein 46’ (RBM46). A highly evolutionarily conserved gene, *Rbm46* is also essential for fertility in both flies and fish. We found *Rbm46* expression was restricted to the mouse germline, detectable in males in the cytoplasm of premeiotic spermatogonia and meiotic spermatocytes. To define its requirement for spermatogenesis, we generated *Rbm46* knockout (KO, *Rbm46*^*-/-*^) mice; although male *Rbm46*^*-/-*^ mice were viable and appeared grossly normal, they were infertile. Testes from adult *Rbm46*^*-/-*^ mice were small, with seminiferous tubules containing only Sertoli cells and few undifferentiated spermatogonia. Using genome-wide unbiased high throughput assays RNA-seq and ‘enhanced crosslinking immunoprecipitation’ coupled with RNA-seq (eCLIP-seq), we discovered RBM46 could bind, via a U-rich conserved consensus sequence, to a cohort of mRNAs encoding proteins required for completion of differentiation and subsequent meiotic initiation. In summary, our studies support an essential role for RBM46 in regulating target mRNAs during spermatogonia differentiation prior to the commitment to meiosis in mice.

## Author summary

Male fertility relies upon continuous daily production of millions of fertilization-competent sperm. These sperm are created in the testis during spermatogenesis, the developmental program founded upon spermatogonial stem cells (SSCs). SSCs divide to produce progeny spermatogonia that either remain in the stem cell pool or commit to differentiate and enter meiosis and ultimately form sperm. The balance between stem cell self-renewal and production of gametes is controlled by changes in the expression of a large complement of genes. An emerging concept in control over gene expression is the essential role of proteins that bind to mRNAs and regulate their stability, storage, and/or translation into proteins. Here, we identify such an RNA binding protein–RBM46 –that is only expressed in the male and female germline and required for gamete production and thus fertility in both sexes. In male mice with a specific deletion of *Rbm46*, spermatogenesis is arrested at spermatogonial differentiation. RBM46 binds a specific cohort of mRNAs encoding factors essential for differentiation and meiosis, and is thus positioned to play a critical role in post-transcriptional control over gene expression in mammalian spermatogonia.

## Introduction

The foundation of mammalian spermatogenesis is provided by the regenerative pool of spermatogonial stem cells (SSCs). SSCs are dispersed throughout the normal testis and, upon division, progeny of SSCs either replenish the SSC pool or proliferate as transit-amplifying undifferentiated progenitor spermatogonia. These progenitor spermatogonia commit to meiosis by differentiating in response to retinoic acid (RA). The essential differentiation program in the mouse lasts 8.6 days, culminating in entry into meiosis as preleptotene spermatocytes. Disruption of spermatogonial fate diminishes male fertility by ultimately impairing sperm production; indeed, a block in differentiation of undifferentiated spermatogonia results in maturation arrest, while overactive differentiation can lead to eventual germline loss. Spermatogonia that commit to the lengthy differentiation program have but two fates–either initiating meiosis as spermatocytes or dying by apoptosis. Indeed, we are unaware of any pharmacologic-treated or mutant or knockout (KO) mouse models with testes containing stable populations of differentiating spermatogonia. Despite the critical nature of the differentiation program, the underlying molecular mechanisms remain largely undefined. One reason for this is the relative paucity of transcriptome-wide changes [1–5]. In line with this, recent studies from our lab revealed RA activates the ‘mammalian target of rapamycin complex 1’ (mTORC1) kinase signaling complex, leading to enhanced translation of differentiation-required proteins such as KIT, STRA8, and SOHLH1/2 [6–9]. Taken together, this reveals a critical reliance upon post-transcriptional control mechanisms for gene regulation during spermatogonial differentiation.

Gene expression can be profoundly controlled at the post-transcriptional level, by regulating pre-mRNA splicing, polyadenylation, mRNA stability, translation, and/or localization [10–12]. These regulatory events are largely directed by sequence-specific RNA binding proteins (RBPs). RBPs are expressed in many tissues and cell types, but male germ cells express an especially high number of unique RBPs. Exemplary germ cell specific RBPs include MSY2, DAZL, BOLL, NANOS2, NANOS3, PIWIL1, DND1, RBMXL2, and DDX4, all of which play essential roles during spermatogenesis, as evidenced by mouse KO studies [13–19]. These RBPs have specialized functions at distinct steps of spermatogenesis, indicating the critical importance of RBPs in regulating gene expression to ensure maintenance of male fertility.

While performing a functional screen for a collection of cDNAs, we observed mRNAs encoding the predicted RBP RBM46 were restricted to testes in mouse and human transcriptome databases [20]. Based on this highly restricted expression pattern, we predicted an essential role for RBM46 in spermatogenesis. To test this hypothesis, we generated *Rbm46* KO (*Rbm46*^-/-^) male mice and discovered loss of RBM46 blocked the completion of spermatogonia differentiation, preventing sperm formation and resulting in infertility. The results presented here position RBM46 as a critical regulator of post-transcriptional gene expression in differentiating mammalian spermatogonia that is essential for completion of spermatogenesis and male fertility.

## Results

### RBM46 is expressed specifically in spermatogonia and spermatocytes in mouse testes

**Fig 1:**
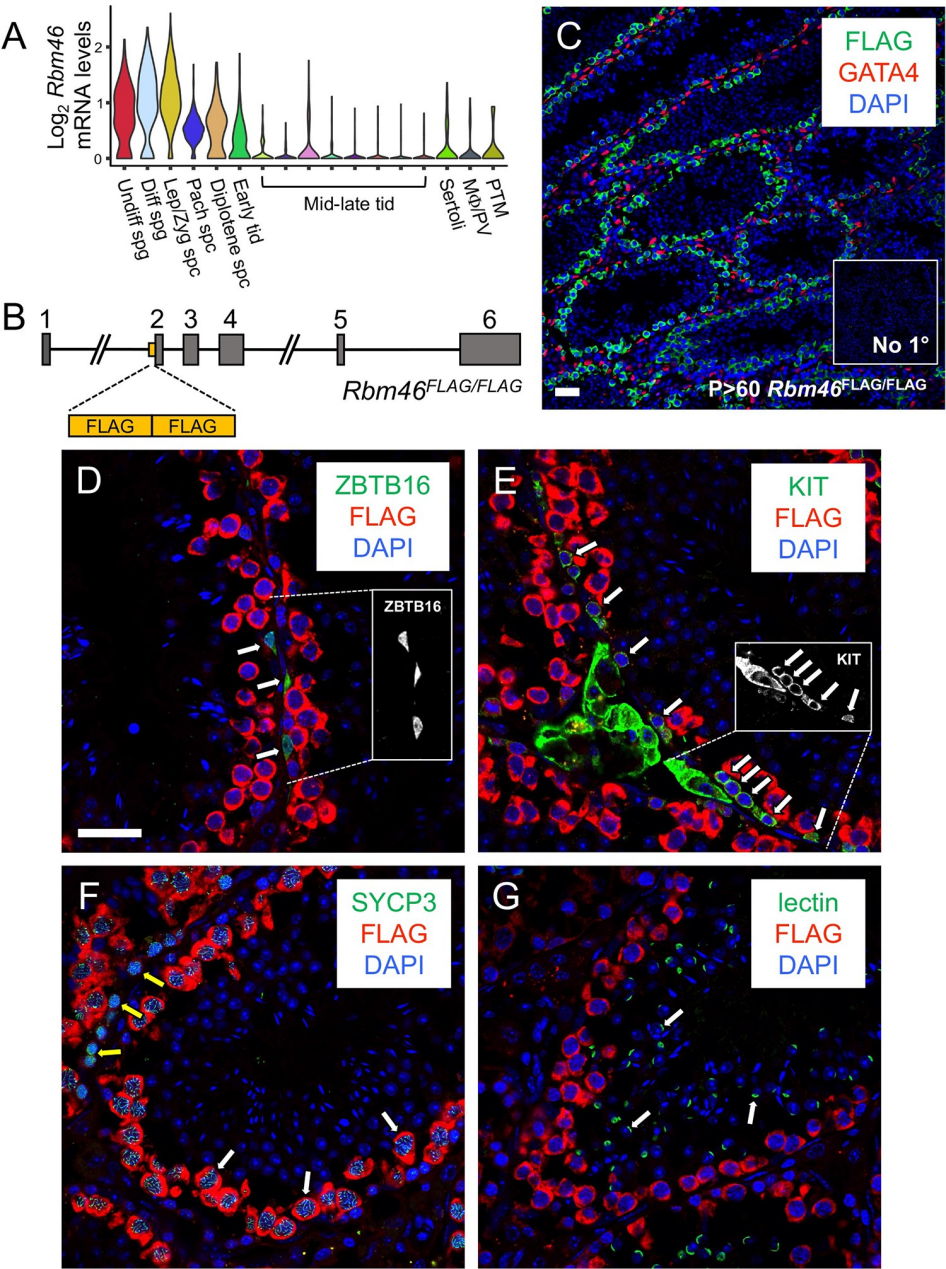
*Rbm46* expression is restricted to spermatogonia, spermatocytes, and early round spermatids in the adult testis. (**A**) Violin plots showing relative mRNA levels from single cell (sc)RNA-seq data from adult mouse testes [4]. Undiff = undifferentiated; diff = differentiating; spg = spermatogonia; lep/zyg = leptotene + zygotene; pach = pachytene; spc = spermatocyte; tid = spermatid; MΦ = macrophage; PV = perivascular; PTM = peritubular myoid. (**B**) Diagram depicting insertion of the 2x FLAG tag upstream of exon 2 of the genomic *Rbm46* locus. (**C-G**) IIF was performed to localize the RBM46-FLAG (green in C, red in D-G) in testes from adult *Rbm46*^*FLAG/FLAG*^ mice. (**C**) RBM46-FLAG (green) was detectable in germ cells but not in GATA4+ (red) Sertoli cells. (**D-G**) RBM46-FLAG (red) was faintly detectable in ZBTB16+ (green) undifferentiated spermatogonia and KIT+ (green) differentiating spermatogonia, indicated by white arrows. Insets in **D-E** are single fluorescent channel images of individual ZBTB16+ undifferentiated and chains of KIT+ differentiating spermatogonia (in white), respectively. RBM46-FLAG (red) became readily detectable in SYCP3+ (green) spermatocytes (white arrows = pachytene, yellow = leptotene) and was undetectable in lectin+ (green) spermatids (white arrows). Nuclei were stained with DAPI (blue). Scale bars = 25 μm.

In a search for novel RBPs expressed in the male germline, we identified a putative candidate encoded by the *Rbm46* gene that was testes-specific in transcriptomic datasets [20]. Analysis of single cell RNA-seq data [21] revealed *Rbm46* mRNAs were detectable in adult testes in undifferentiated and differentiating premeiotic spermatogonia, increased in preleptotene, leptotene/zygotene, and pachytene meiotic spermatocytes as well as secondary spermatocytes, declined in early postmeiotic round spermatids, and were undetectable in mid- and late round spermatids, as well as somatic cells of the testis (Fig 1A). We next sought to define the expression pattern of RBM46 protein in mouse testes. Since none of the commercially available antibodies yielded consistent results in immunostaining, CRISPR/Cas9 technology was used to generate mice with tandem copies of the FLAG epitope tag inserted at the N-terminus of RBM46 (Fig 1B). Male mice with homozygous insertion of sequences encoding the FLAG tag (*Rbm46*^*FLAG/FLAG*^) appeared normal and were fertile; their histologically normal testes (S1 Fig) suggested the FLAG tag did not adversely affect RBM46 function. Immunostaining these adult testes using anti-FLAG antibodies revealed RBM46 protein was specifically expressed in cytoplasm of undifferentiated and differentiating spermatogonia as well as spermatocytes, but not in spermatids, sperm, or somatic cells (Fig 1C–1G).

### RBM46 is essential for fertility in both sexes

**Fig 2:**
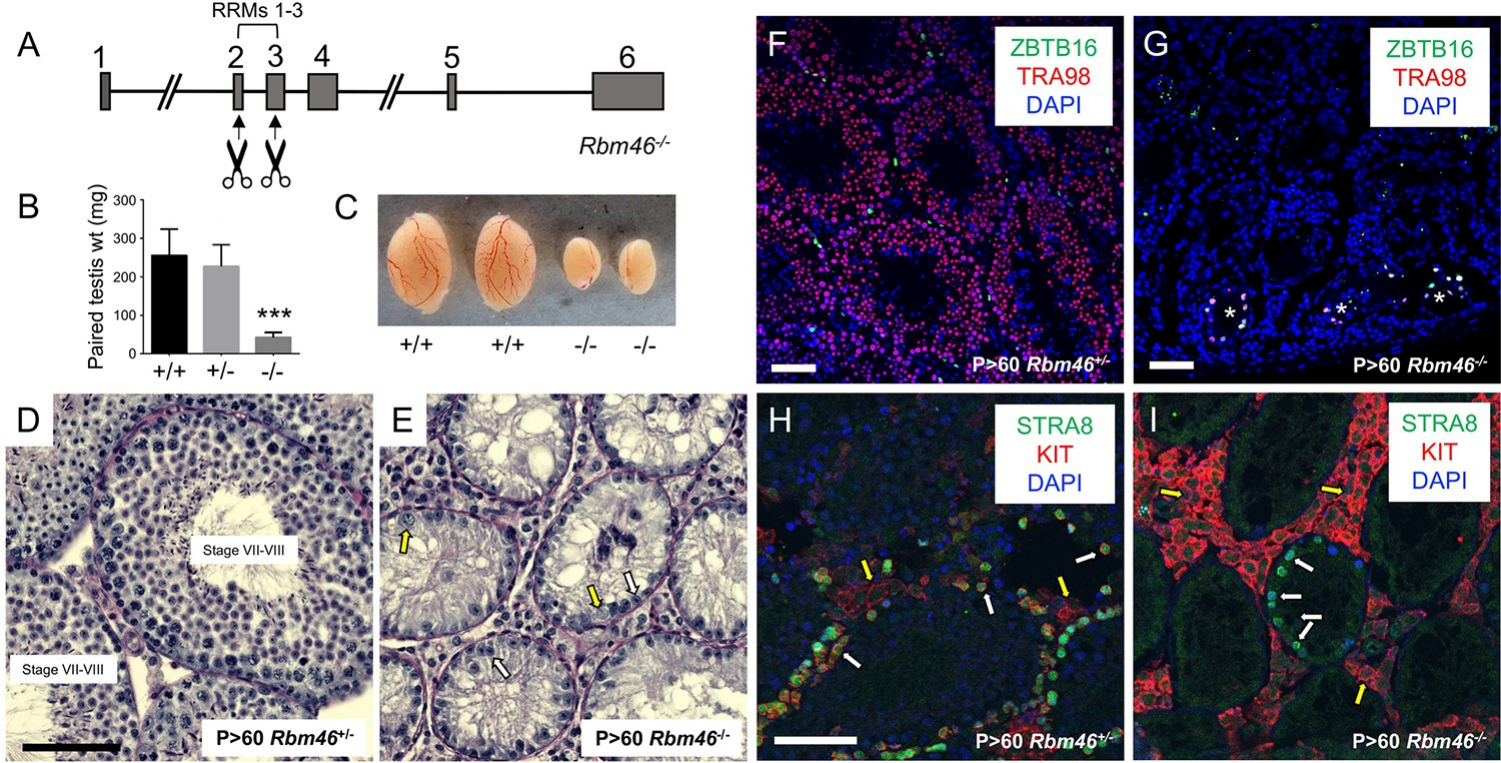
Adult *Rbm46*^-/-^ testes were dramatically reduced in size and contained only spermatogonia. (**A**) Diagram showing the genomic *Rbm46* locus and the cut sites for deletion within exons 2–3. (**B-C**) Testes from *Rbm46*^-/-^ mice were significantly smaller than those from *Rbm46*^+/+^ littermate controls. (**D-E**) PAS-stained Bouin’s-fixed testes from adult *Rbm46*^+/-^ (**D**) and *Rbm46*^-/-^ (**E**) mice, respectively. In contrast to control (**D**), *Rbm46*^-/-^ seminiferous epithelia (**E**) exhibited a single cellular layer, which included apparent Sertoli cells (white arrows) and spermatogonia (yellow arrows). (**F-I**) IIF was done to confirm the cellular identity of remaining cells in *Rbm46*^-/-^ seminiferous epithelia. Compared to controls (**F**), *Rbm46*^-/-^ seminiferous epithelia (**G**) contained ZBTB16+ (green) undifferentiated spermatogonia (in tubules marked with an asterisk), and germ cells were immunostained for the pan germ cell marker TRA98 (red). Compared to controls (**H**), some spermatogonia (indicated by white arrows) in *Rbm46*^-/-^ testes were STRA8+ (green, **I**), indicating response to RA, but none were KIT+ (red, **I**), revealing impaired differentiation. Interstitial cells (marked by yellow arrows, shown in **H-I**) are always KIT+. Nuclei were stained with DAPI (blue). Triple asterisks indicate statistical significance at P<0.001. Scale bars = 50 μm.

To define the requirement for the RNA binding protein RBM46 in spermatogenesis, CRISPR/Cas9 technology was used to generate *Rbm46*^*-/-*^ mice. A founder male was identified with a frameshifting deletion between exons 2–3 (Fig 2A). This frameshift in the region encoding the first RNA Recognition Motif (RRM) led to a premature termination codon that disrupted all three consensus RRMs, giving high confidence for a functional null allele (S2A–S2C Fig). *Rbm46*^*-/-*^ mice were viable, healthy, and displayed no overt defects (not shown). However, neither *Rbm46*^*-/-*^ male nor female mice were able to produce pups when mated with WT counterparts, revealing a requirement for RBM46 in fertility. Compared to WT littermates, adult *Rbm46*^*-/-*^ ovaries lacked oocytes, revealing complete loss of the germline (S3A and S3B Fig).

In this study, we focused on the male infertility phenotype. Paired testis weights of *Rbm46*^*-/-*^ mice were considerably lower (42.5 ± 13.0 mg) than those from *Rbm46*^*+/+*^ (255.5 ± 67.9 mg) and *Rbm46*^*+/-*^ (227 ± 55.9 mg) littermate controls (Fig 2B). This dramatic decrease in *Rbm46*^*-/-*^ testis size (Fig 2C) suggested impaired spermatogenesis. Indeed, histological analysis confirmed that, as compared to *Rbm46*^*+/+*^ and *Rbm46*^*+/-*^ testes (which appeared normal, e.g., Fig 2C and 2D), *Rbm46*^*-/-*^ testes had severe defects in spermatogenesis, with seminiferous tubules containing Sertoli cells and only a few apparent spermatogonia, but lacking spermatocytes, spermatids, or testicular sperm (Fig 2E). This result was confirmed by staining adult testes for the pan germ cell marker TRA98 (also termed GCNA [18, 22, 23] along with the somatic Sertoli cell marker GATA4 (S4A and S4B Fig). In comparison to normal-appearing *Rbm46*^*+/-*^ testes (Fig 2F), most tubules in *Rbm46*^*-/-*^ adult mice lacked germ cells, although there were isolated populations of ZBTB16+/TRA98+ undifferentiated spermatogonia (Fig 2G). To confirm the absence of more advanced germ cells in *Rbm46*^*-/-*^ testes, co-immunostaining was done to detect differentiating spermatogonia markers KIT and STRA8, the latter of which is also highly expressed in preleptotene spermatocytes [24, 25]. As expected, tubule cross sections in control testes contained numerous KIT+ differentiating spermatogonia and STRA8+ preleptotene spermatocytes (Fig 2H). In *Rbm46*^*-/-*^ testes, some TRA98+ spermatogonia were also STRA8+, revealing the capacity to respond to RA; however, none were KIT+ (Figs 2I and S4F), suggesting an inability to undergo bona fide RA-induced differentiation. In agreement with this, there were no SYCP3+ meiotic spermatocytes in *Rbm46*^*-/-*^ testes, in contrast to controls (S4C–S4F Fig). Taken together, these findings suggest impaired spermatogonial differentiation and reveal an absence of meiotic spermatocytes in *Rbm46*^*-/-*^ testes.

Although *Rbm46* mRNA and protein were detectable primarily in germ cells, we tested the cell-autonomous requirement by generating germ cell-specific conditional KO mice. These mice were created by crossing *Rbm46*^*fl/fl*^ and *Stra8-iCre*, the latter of which is expressed beginning in undifferentiated progenitor spermatogonia [26]. The testis phenotype of adult *Rbm46*^*fl/-*^*;Stra8-Cre* mice (S5 Fig) was indistinguishable from those with conventional whole-body deletion (Fig 2), confirming an essential cell autonomous role for RBM46 during male germ cell development.

### Spermatogonial differentiation is impaired in developing Rbm46^-/-^ testes

**Fig 3:**
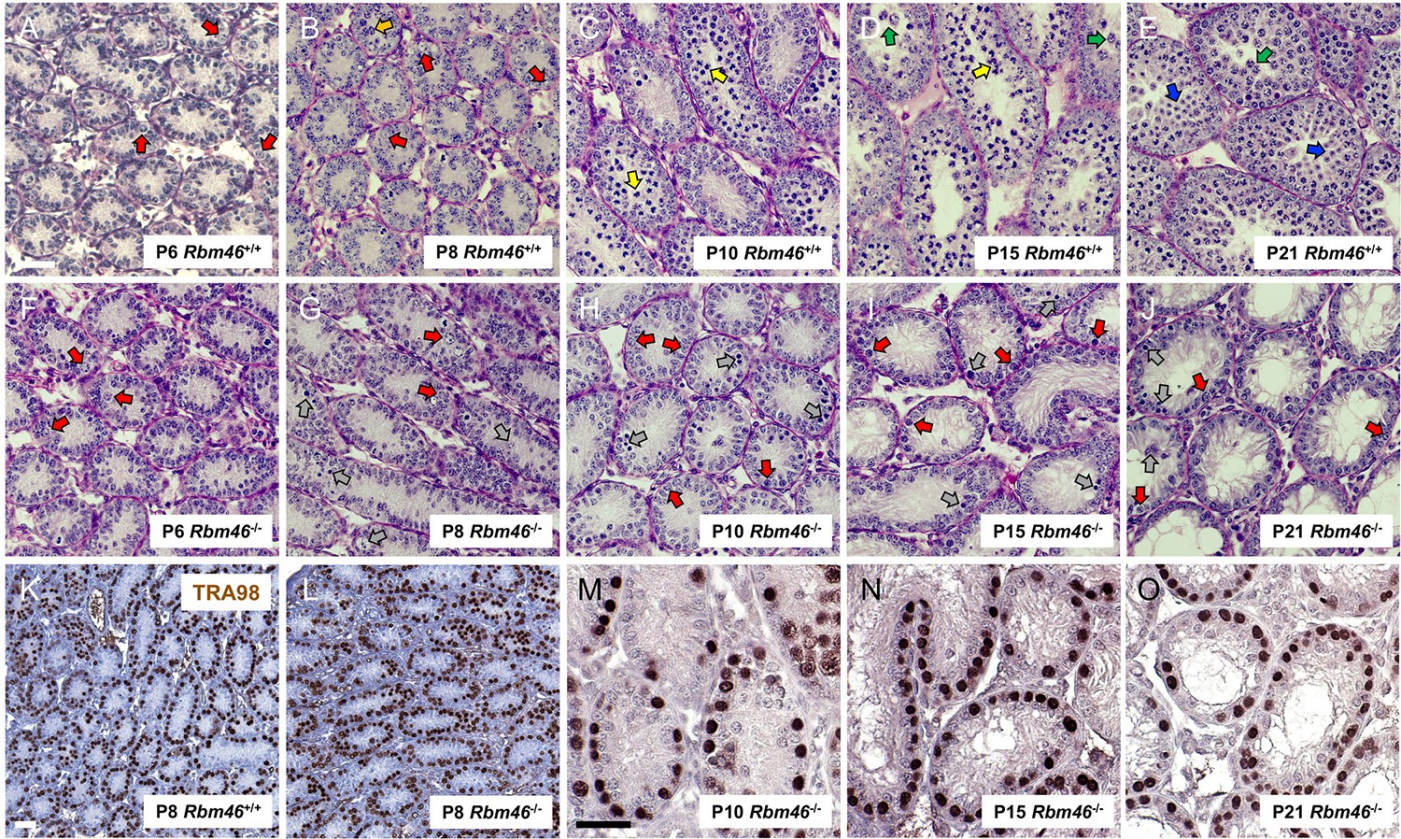
Germ cell loss was evident after P8 during the first wave of spermatogenesis in *Rbm46*^*-/-*^ testes. (**A-E**) The first cohort of spermatogenic cells appear on predictable days during the first wave of spermatogenesis in control PAS-stained *Rbm46*^+/+^ testes. These include spermatogonia (red arrows), preleptotene spermatocytes (orange arrows), leptotene spermatocytes (yellow arrows), pachytene spermatocytes (green arrows), and round spermatids (blue arrows). (**F-J**) In contrast, *Rbm46*^-/-^ testes contained only spermatogonia (red arrows) and apparently degenerating germ cells (grey arrows). Meiotic spermatocytes were not observed at any age examined. (**K-L**) P8 testes contain abundant TRA98+ (brown) spermatogonia in both control and *Rbm46*^-/-^ testes. (**M-O**) P10, P15, and P21 *Rbm46*^-/-^ testes contained TRA98+ (brown) basally located spermatogonia. Scale bars = 50 μm.

We next sought to precisely define the onset of the spermatogenic defect in *Rbm46*^*-/-*^ testes. To accomplish this, we examined *Rbm46*^*-/-*^ testes during the well-characterized first wave of spermatogenesis, when populations of progressively advanced germ cells predictably appear on successive days [27]. In control testes, at P6, 8, 10, 15, and 21 the most advanced germ cell types were differentiating spermatogonia, preleptotene spermatocytes, leptotene spermatocytes, pachytene spermatocytes, and round spermatids, as expected [28] (Fig 3A–3E). In stark contrast, *Rbm46*^*-/-*^ testes only contained apparent spermatogonia on each of these days (Fig 3F–3J, 3K and 3L), and there was no difference in numbers of spermatogonia as early as P6 (S6 Fig).

**Fig 4:**
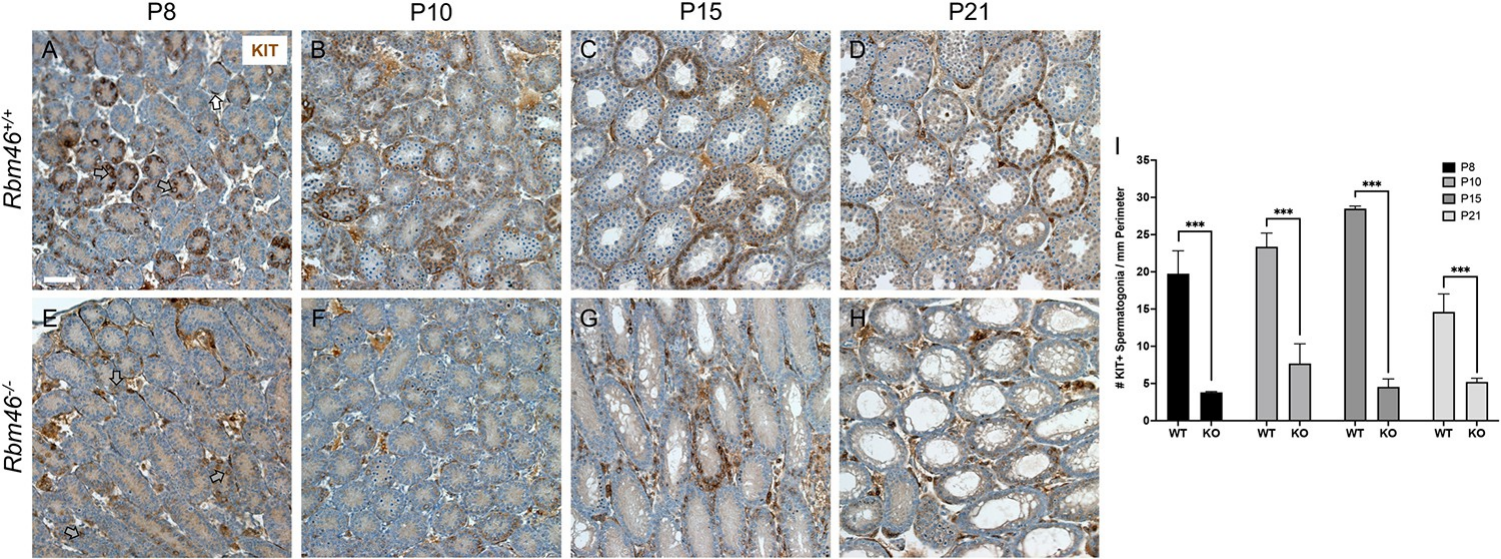
KIT+ differentiating spermatogonia were reduced dramatically in developing *Rbm46*^-/-^ testes. (**A-D**) Darkly staining KIT+ (brown) spermatogonia (gray arrows, **A**) were numerous in *Rbm46*^+/+^ testes. KIT+ interstitial cells are indicated with white arrows. (**E-H**) At P8, there are scattered faintly staining KIT+ spermatogonia (gray arrows, **E**), and fewer are seen afterwards, from P10 (**F**) through P15 (**G**) and then at P21 (**H**). (**I**) Quantitation of KIT+ spermatogonia at each age. Scale bar = 50 μm. Triple asterisks indicate statistical significance at P<0.001.

To confirm the identity of the resident germ cells in developing *Rbm46*^*-/-*^ testes, we performed immunostaining for the bona fide spermatogonia differentiation protein marker KIT, which also is expressed in somatic cells in the interstitial compartment [29–34]. At P8, 10, 15, and 21 KIT was readily detectable in the membrane of differentiating spermatogonia, as expected (Fig 4A–4D). In *Rbm46*^*-/-*^ testes, significantly fewer KIT+ spermatogonia were present at each of these ages, with numbers remaining stagnant as the mice age (Fig 4E–4I). Thus, we conclude that although spermatogonia initiated the program of differentiation, it was not sustained, leading to stalled germ cell development and an absence of meiotic cells.

### RBM46 is required for activation of differentiation- and meiosis-associated gene expression in spermatogonia

**Fig 5:**
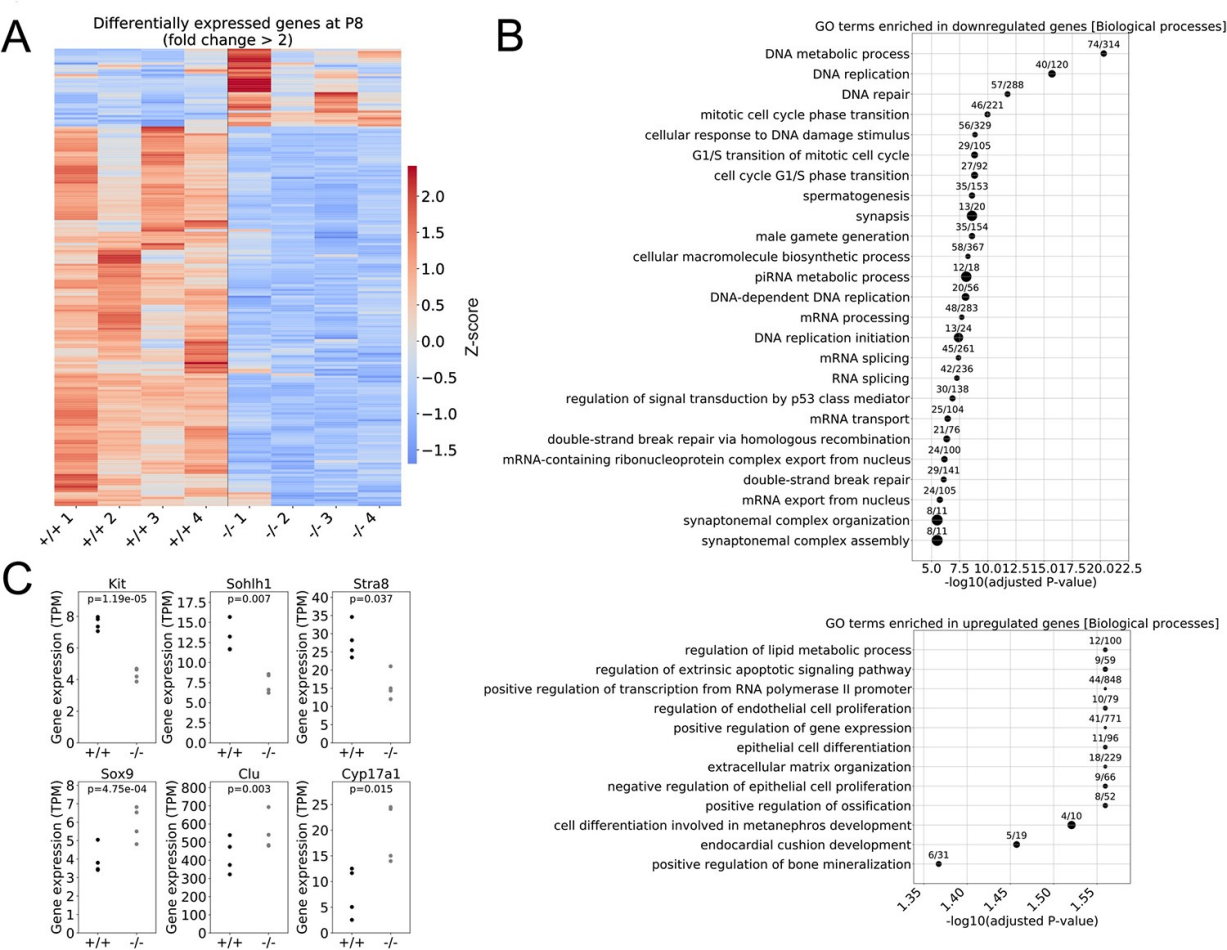
Genes involved in cell cycle regulation were deregulated in *Rbm46*^*-/-*^ testes at P8. (**A**) Heatmap of 200 genes with >2-fold changes in *Rbm46*^-/-^ relative to *Rbm46*^*+/+*^ controls at P8. (**B**) Gene ontology terms for biological processes enriched in genes that are downregulated (top) or upregulated (bottom) in *Rbm46*^*-/-*^. Circle size and numbers correspond to the number of genes that are differentially expressed and represented in a GO term over to the total number of genes listed in the GO term. (**C**) Expression level of genes involved in spermatogonia differentiation and somatic cell markers. P-values are DESeq2 adjusted p-values comparing *Rbm46*^*+/+*^ to *Rbm46*^*-/-*^ testes.

To begin to define underlying molecular defects in *Rbm46*^*-/-*^ spermatogonia, we performed bulk RNA-Seq on WT and *Rbm46*^*-/-*^ testes from P8 mice. This age was selected for analysis as it represented a time that, although there was some germ cell degeneration in *Rbm46*^*-/-*^ testes (Fig 3G), WT and *Rbm46*^*-/-*^ testes had similar apparent numbers of germ cells (Fig 3K–3L). Quantitation revealed a ~21% decrease in numbers of TRA98+ germ cells in *Rbm46*^*-/-*^ testes. We reasoned differences in gene expression at the mRNA level would reveal key dysregulated genes due to either direct regulation by RBM46 on mRNA stability or indirect downstream consequences of *Rbm46* deletion. We used DESeq2 to identify differences in mRNA levels between WT and *Rbm46*^*-/-*^ testes. For protein coding genes (using a cutoff adjusted p-value <0.05), we identified 561 upregulated and 1,218 downregulated transcripts (S1 Table). Changes in mRNA abundance were modest, with only 167 downregulated genes and 33 upregulated genes showing >2-fold changes (Fig 5A). Gene ontology (GO) analysis of downregulated genes identified numerous terms relevant to spermatogenesis, including several related to meiosis: ‘*spermatogenesis*’, ‘*synapsis*’, ‘*male gamete generation*’, and ‘*synaptonemal complex assembly*’ (Fig 5B). Examples of meiotic genes with reduced mRNA levels in *Rbm46*^*-/-*^ testes included *Dmc1*, *H2afx*, *Meiob*, *Spo11*, *Mov10l*, *Hormad1*, *Sycp2*, and *Sycp3*. We also identified reduced levels of several mRNAs encoding proteins involved with (e.g., *Stra8*) or required for (e.g., *Kit*, *Sohlh1*; Fig 5C) spermatogonia differentiation. There were no significant changes in mRNA levels of most markers of undifferentiated spermatogonia (e.g., *Gfra1*, *Id4*, *Nanos2/3*, *Cdh1*, *Ret*, *Itga6*, *Itgb1*, and *Sall4*). GO analysis of upregulated genes did not identify terms with apparent relevance to spermatogenesis (Fig 5B). We did, however, note increased levels of somatic cell markers (e.g., Sertoli cell mRNAs *Sox9* and *Clu* and Leydig cell markers *Cyp17a1*, *Hmgcs2*, and *Prlr* [21, 35, 36]). Using the Majiq computational pipeline [37], we only found few changes in alternative splicing (see S2 Table) and, although the splicing differences were important (S7A Fig) and mostly involved alternative first or last exon events (S7A Fig), all but eight genes (*Lrif1*, *Apobec3*, *Zfp429*, *Chd1l*, *Prickle2*, *Selenbp2*, *Zfp697*, *Ndufs1*) affected at the splicing level were unaffected at the level of mRNA abundance. Genes with differential splicing were not enriched for any specific GO term. In summary, there was an apparent decrease in the mRNA abundance of genes encoding proteins required for spermatogonial differentiation and meiosis, which is likely due to indirect action of RBM46, in that differentiating spermatogonia and preleptotene spermatocytes were absent in P8 *Rbm46*^*-/-*^ testes.

### RBM46-bound mRNAs are enriched for functions in RNA processing, meiosis, and translation regulation

**Fig 6:**
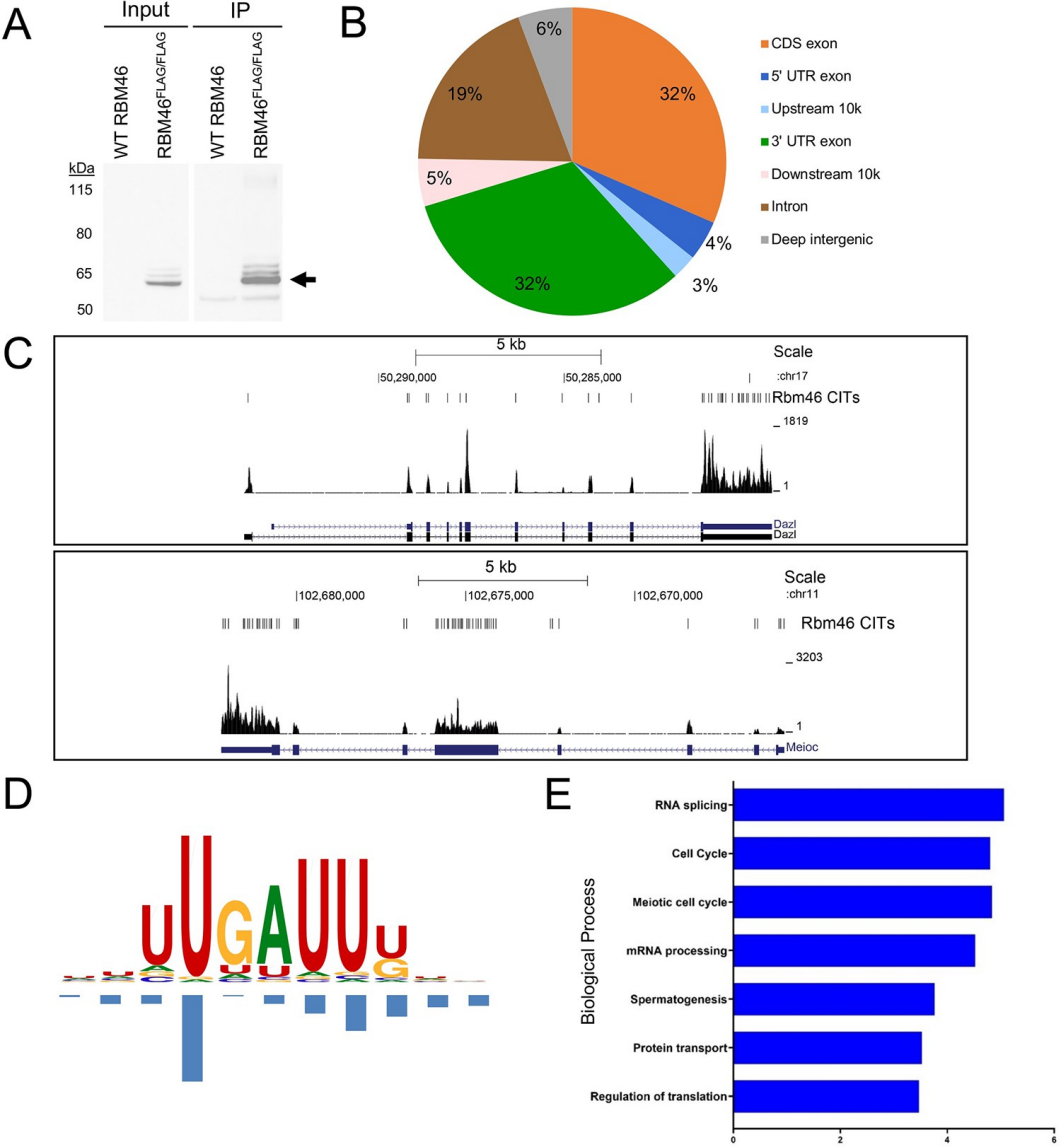
RBM46 sequence-specific binding to a specific cohort of mRNAs encoding factors required for RNA processing and meiosis. (**A**) FLAG-stained western blot of lysates from *Rbm46*^*WT/WT*^ and *Rbm46*^*FLAG/FLAG*^ showing input and immunoprecipitated proteins, respectively. Band at the expected protein size is indicated by arrow. (**B**) Genomic distribution of RBM46 binding in the testis, displayed as percentages of binding to each sequence feature. (**C**) Representative UCSC genome browser view showing exonic binding of RBM46 across *Dazl* and *Meioc*. (**D**) WebLogo showing U-rich RNA binding sequence motif identified using mCross as the most enriched sequence at the RBM46 crosslink sites. (**E**) Gene ontology analysis performed using EnrichR showing the top biological processes of RBM46-bound mRNAs.

To identify mRNAs directly bound by RBM46 in the male germline, we used enhanced crosslinking coupled with immunoprecipitation and RNA-seq (eCLIP-Seq). This method provides unbiased genome-wide coverage from small amounts of cellular input, enabling identification of RBP binding sites at single nucleotide resolution [38]. We used testes from *Rbm46*^*FLAG/FLAG*^ mice (Fig 1B), as the FLAG-tagged RBM46 protein can be efficiently and specifically immunoprecipitated using FLAG antibodies. Because RBM46 is expressed in both spermatogonia and spermatocytes (Fig 1D–1F), we used eCLIP in testes from *RBM46*^*FLAG/FLAG*^ mice at P21, an age when they contain spermatogonia, spermatocytes, and the very first emergent round spermatids [28]. Immunoprecipitated material was separated by electrophoresis, transferred to a nitrocellulose membrane, and the region containing crosslinked RNAs excised and released from the membrane (Figs 6A and S8). eCLIP libraries were prepared and five replicate eCLIP samples were sequenced with corresponding inputs, processed, and mapped at ~8 x 10^6^ non-redundant reads to the genome (mm10) [39]. We anticipated enrichment of binding sites in mRNA 3’ untranslated regions (3’ UTRs), similar to reports of other cytoplasmic RBPs in male germ cells [40–43]. To our surprise, nearly equal percentages of CLIP tags were present in the 3’ UTR and protein coding sequences, though when corrected for the percentage of these regions in the transcriptome there was a modest enrichment of binding sites in the 3’ UTR over the CDS (Fig 6B). CLIP tags also showed a relatively uniform distribution across mRNAs (Fig 6C).

To determine the binding specificity of RBM46, we extracted sequences around CITS and performed *de novo* motif discovery using mCross, an algorithm developed to simultaneously model RBP binding specificity and the crosslink position in the binding motif [44]. After pooling all replicates, mCross was used extract sequences around crosslink-induced truncation sites for *de novo* motif discovery, which identified 90,243 crosslink-induced truncation sites (CITS, P<0.001) [39, 45]. This analysis revealed a U-rich motif with a UGAU core and predominant crosslinking at the U1 position of the core (Fig 6D). The UGAU motif is highly enriched at the crosslink sites (with a 25-fold enrichment for crosslinking at U1 of the UGAU motif), while a moderate enrichment was observed in regions around CLIP tag peaks. Given the high signal-to-noise ratio of CITS, we identified a stringent subset of RBM46 target transcripts based on the presence of CITS satisfying two criteria: 1) presence of the UGAU motif with crosslinking at the U1 position; and 2) ≥50 putative truncated tags at the crosslink sites. This allowed us to identify 1,349 CITS associated with 873 unique genes. Gene Ontology (GO) analysis of these genes was performed using DAVID [46]. RBM46 target transcripts were enriched for terms relevant to spermatogenesis using GO analysis (Fig 6E). Of note, there was significant enrichment of genes involved in RNA processing that included several RBPs with functions in spermatogenesis (e.g., DAZL, BOLL, PABPC1, CELF1, CEBP1, PTBP2, and RBM46 itself) and translation initiation factors (e.g., EIF1A, EIF2S1, EIF4G1, and EIF4G2). RBM46 also showed enriched binding to mRNAs encoding essential meiosis proteins (e.g., SYCP1, SYCP2, SYCP3, MEIOC, SPO11, TEX15, HORMAD1, HSPA2, and BRCA2). A list of mRNAs that were bound by RBM46 at P21 and exhibited differential abundance in P8 *Rbm46*^*-/-*^ testes are presented in S3 Table.

## Discussion

Here, we localized the germ cell specific RBP RBM46 to the cytoplasm of spermatogonia and spermatocytes, but not in other testes germ cell types nor in somatic cells. We generated KO mice and discovered a germ cell autonomous requirement for RBM46 in spermatogenesis and male fertility. Specifically, RBM46 was essential, in spermatogonia, to complete differentiation in both developing and adult testes. *Rbm46* KO testes had altered transcriptomes, with downregulation of transcripts encoding differentiation- and meiosis-associated genes. Using enhanced crosslinking immunoprecipitation [38] followed by binding analysis with the CLIP Tool Kit [39], we determined RBM46 directly bound, at a U-rich consensus sequence, to mRNAs encoding proteins involved in spermatogenesis as well as in general translation regulation. In summary, RBP46 is required for spermatogonial differentiation and male fertility, and directly binds to mRNAs encoding genes essential for differentiation and meiosis in the male germline.

All stages of spermatogenesis, from survival of prospermatogonia to the maintenance of SSCs to meiosis and spermiogenesis, require post-transcriptional regulation by RBPs. Indeed, a number of essential RBPs have been identified that repress or activate the translation of select mRNAs, including NANOS2, NANOS3, DAZL, TIAR/TIAL1, PIWIL2/MILI, PIWIL4/MIWI2, DDX4/VASA, MSY2, and LIN28A [16, 40, 43, 47–52]. The functions of essential RBPs include regulation of mRNA splicing, polyadenylation, localization, stability/degradation, and translation [53–55]. While the mechanistic functions and global regulatory targets have been identified for multiple essential RBPs, many RBPs necessary for spermatogenesis remain to be defined and characterized. Therefore, identification of a novel RBP and its genome-wide regulatory targets provides new insights into the molecular pathways that control germ cell gene expression during maintenance and differentiation.

Once in the cytoplasm, mRNAs face three possible fates: translation, storage, or degradation. Transit between these fates is well-known to regulate key transitions during male germ cell development [56, 57]. Our RNA-Seq findings offer further support for a spermatogonial differentiation block in *Rbm46*^*-/-*^ testes. However, the changes in mRNA levels were rather modest, including markers of differentiated spermatogonia and meiotic genes. The RNA-seq experiment was performed using testes at P8, a time when there was a ~21% decrease in germ cells, notably differentiating spermatogonia and the first emergent preleptotene spermatocytes entering meiosis. The loss of these cells, and their transcriptomes suggest many, if not most changes in transcript levels in *Rbm46*^*-/-*^ testes were indirect, due to the differentiation impairment and not due to changes in RNA posttranscriptional control.

RBM46 was recently discovered to be part of a complex containing several essential proteins. These include the disordered protein MEIOC (required for mouse meiosis [58, 59]), the exoribonuclease XRN1 (Drosophila *pacman*, required for spermatogenesis and male fertility [60]), and the RNA helicase YTHDC2 (Drosophila *bgcn* [61], required for progression through meiosis in the male mouse germline [62–66]). This YTHDC2-containing complex bound in testes containing both spermatogonia and spermatocytes to a canonical U-rich binding motif [67, 68]. This sequence closely resembles the one identified here, in P21 testes, which contain spermatogonia, spermatocytes, and the first emergent spermatids [28]. YTHDC2’s function in gametogenesis was recently shown to be independent of its N6-methyladenosine (m6A)-modified RNA binding [67, 68]; therefore, it is possible that RBM46, as a resident in this RNA management complex, provides additional RNA binding function (allosterically or directly) through the U-rich binding sequence we identified. In Drosophila, Bgcn is required for translation control and expressed in a reciprocal pattern to the Nanos proteins [69]. Based on published reports and the present data, this arrangement appears to be conserved in mice–we discovered a requirement for RBM46 in spermatogonial differentiation, whereas others have shown NANOS2 and NANOS3 are required for SSC maintenance [70–72]. Therefore, RBM46 may aid in target recognition for YTHDC2 functions in translational regulation.

The uniform binding observed across both coding sequences and UTRs of mRNA transcripts is somewhat uncommon among RBPs but resembles the diffuse mRNA binding pattern shown by CLIP of Fragile X mental retardation protein (FMRP) and LIN28A [51, 73]. FMRP is present in actively translating polysomes and regulates translation [74–76]. Similarly, binding of LIN28A across the CDS and UTRs positively regulates translation of mRNAs, including meiotic transcripts in mouse testes [52, 77–79]. Thus, the atypical binding pattern of RBM46 is consistent with or permissive for a role in translation regulation.

RBM46 is a highly conserved RBP whose function has been examined in flies, fish, and now mice. In Drosophila, the mouse ortholog of RBM46 is encoded by the RBP ‘tumorous testis’ (Tut), which is required for spermatogenesis and male fertility [80]. Interestingly, the phenotype of Tut mutant flies is similar to that reported here–germ cell development is blocked at differentiation, and thus contain only undifferentiated spermatogonia. In addition, in zebrafish (*Danio rerio*), *rbm46* is expressed in male germ cells and is required for spermatogonia to enter or progress through meiosis. [81]. Indeed, male *rbm46* mutants were sterile, with testes containing only spermatogonia that proliferated into >16 interconnected 3C-4C germ cells, suggesting incorrect meiotic entry. *Rbm46*-depleted gonads were sex-reversed to testes, and transcriptome analyses revealed many more changes in mRNA abundance (4,436 up and 3,571 down) than we observed here, including reduced levels of many meiotic mRNAs (e.g., *spo11*, *dmc1*, *rad51*, *msh4*, *mlh1*, *rec8*, *smc1b*, *sycp1-3*). These findings support a major role in directing meiotic gene expression. Here, we identified numerous mRNAs encoding essential meiosis proteins among the top RBM46 CLIP targets in P21 testes containing a mixture of spermatogonia and spermatocytes (e.g., SYCP1, SYCP2, SYCP3, MEIOC, SPO11, TEX15, HORMAD1, HSPA2, and BRCA2). These findings provide further support for RBM46 functions in meiosis while also suggesting that mRNAs highly bound by RBM46 support translation, or at least do not inhibit it.

In two previous studies from the same research group, a critical role for RBM46 was reported in embryonic stem cell (ESC) and trophectoderm differentiation [82, 83]. These studies found RBM46 promoted *Cdx2* mRNA stability and degradation of beta-catenin (*Ctnnb1*) mRNAs in ESCs. However, the second manuscript was recently retracted by the authors [84]. It is notable that *Rbm46* expression is rather low in ESCs in available datasets, suggesting the primary roles of RBM46 are in male germ cell development and function. Furthermore, the fact that male *Rbm46*^*-/-*^ mice were otherwise normal, without any phenotypes other than infertility, is not compatible with an essential role of RBM46 outside of the germline.

## Methods

### Ethics statement

All animal procedures and experiments were approved by the Institutional Animal Care and Use Committees (IACUC) at the University of Pennsylvania (protocol #803164) and East Carolina University (approval A3469-01).

### Mouse strains

*Rbm46*^*FLAG/FLAG*^ mice and *Rbm46*^*-/-*^ mice were generated in the Penn Transgenic and Chimeric Mouse and CRISPR-Cas9 Mouse Targeting Core Facilities (supported by NIH grant P30DK050306). To create *Rbm46*^*FLAG/FLAG*^ mice, Alt-R CRISPR-Cas9 crRNA (Integrated DNA Technologies (IDT: Iowa City, IA)) targeting the sequence 5’-ATCAGTGTTTCTTCATTCA-3’ (anti-sense) and a rescue donor oligo were created containing two tandem copies of the FLAG Tag in-frame after the ATG start codon with a 5’ 91 nt homology arm and 3’ 37 nt homology arm. The crRNA and donor oligos were microinjected in fertilized eggs together with an mRNA encoding Cas9 protein.

For *Rbm46*^*-/-*^ mice, two crRNAs were generated *in vitro* using T7 polymerase to target the following sequences in *Rbm46* exon 2 (5’- ATGAATGAAGAAAACACTGA-3’ and 5’-ATAATTGTTAAGAATCCGGA-3’ (anti-sense)). The two crRNAs were microinjected together into fertilized eggs along with Cas9 mRNAs. Resulting pups were screened by PCR for heterozygous KI or deletion and founder mice were confirmed by DNA sequencing. Mice were humanely euthanized by CO_2_ asphyxiation followed by cervical dislocation. Mice were on a B6SJLF1/J hybrid genetic background (strain #100012, The Jackson Laboratory).

### Tissue collection, fixation, and immunostaining

For cryosections or paraffin embedding, testes were fixed for 4 hrs–overnight in either fresh 4% paraformaldehyde or Bouin’s solution, respectively, at 4°C and prepared as described previously [85]. Bouin’s-fixed testes were stained with Periodic Acid Schiff (PAS) using standard methods. For immunohistochemistry (IHC), immunostaining was performed on Bouin’s-fixed sections as described [85]. Brightfield images were captured on an Axio Observer A1 inverted microscope outfitted with a Zeiss Axiocam 503 color digital camera and Zen software (Carl Zeiss Microscopy, LLC).

For indirect immunofluorescence (IIF), immunostaining was performed on cryosections as described [85]. Alexa-Fluor conjugated secondary antibodies (Thermo Scientific) raised against the animal host of the primary antibody (Table 1) were incubated for 1 hr at room temperature at a 1:500 dilution. Coverslips were mounted for IIF with Vectastain containing DAPI (Vector Laboratories). Sections were imaged using a Fluoview FV1000 confocal laser scanning confocal microscope (Olympus America).

**Table 1:** Antibodies and immunostaining reagents.

*Antigen*	*Host*	*Source*	*Dilution*	*Catalog number*
TRA98	rat	Abcam	1:1000	ab82527
ZBTB16/PLZF	Goat	R & D Systems	1:1000	AF2944
KIT	Goat	R & D Systems	1:1000 (IIF); 1:500 (IHC)	AF1356
SYCP3-488	Mouse	Abcam	1:200	Ab205846
FLAG	Rat	Novus	1:250	NBP1-06712SS
STRA8	Rabbit	Abcam	1:3000	ab49602
GATA4	Rabbit	Cell Signaling Technology	1:400	36966S
Lectin-488	Peanut	ThermoFisher Scientific	1:500	L21409

### RNA-seq

Testes from P8 mice were flash frozen in liquid nitrogen and ground using a mortar and pestle. Ground tissue was homogenized in TriZol reagent by passing samples through 18- and 26-gauge needles, and RNA was extracted with RNeasy minikit (Qiagen) using manufacturer’s instructions. Total RNA was then submitted to Genewiz and Illumina libraries were prepared after rRNA depletion using the Illumina Ribo-Zero kit. Sequencing was performed using Illumina HiSeq for 150 bp paired end sequencing using four replicates each from wild type control and *Rbm46*^*-/-*^ samples. Adapters were trimmed from RNA-Seq samples using BBDuk, aligned to the mouse GRCm38 genome assembly using STAR v.2.5.1B, and sorted and indexed using samtools v.1.9. For gene expression quantification, salmon v.0.14.0 was used in mapping-based mode with selective alignment on trimmed fastq files using GENCODE vM23 annotation to create the index. Differential gene expression analysis was performed with DESeq2 v.1.22.2. Differential splicing analysis was performed with MAJIQ v.2.1 using GENCODE vM23 reference transcriptome annotation without intron retention quantification. We identified differentially spliced junctions by keeping junctions that had a delta PSI of at least 15 with a probability that the delta PSI is above 15 of at least 95%. Gene ontology analysis was performed with enrichR v.1.0 using a 2018 release of the GO Consortium annotations.

### eCLIP-seq

Testes were harvested from mice and rinsed in PBS. Testes were detunicated, triturated, dounced in PBS, and tissue material was crosslinked three times at 400 mJ/cm^2^ using a Stratalinker 2400 (Stratagene). Samples were then flash-frozen in liquid nitrogen and stored at -80°C until use. Each replicate was derived from a pair of testes from a single mouse. Samples were lysed, and crosslinked RNP complexes were treated with 5 U/ml RNAse I, immunoprecipitated, and used to generate eCLIP libraries and control input libraries as previously described [38]. In brief, to extract RBM46-specific interactors, cleared immunoprecipitants were resolved on 4–12% Bis-Tris protein gel and transferred to a nitrocellulose membrane. The RNA:RNP complex was extracted from the nitrocellulose membrane by cutting a region that included the RNA binding protein, RBM46 (size ~62 kDa) and a region of the membrane ~50 kDa above the RBM46 band. The RNA was isolated from the membrane following proteinase K and urea treatments. An Illumina Nova-Seq was used for 50 bp paired end sequencing. Raw data from *Rbm46* eCLIP experiments and input controls were processed using CLIP Tool Kit (CTK) [39]. Unique tags were identified after stringent mapping to the reference genome (mm10) and collapsing of PCR duplicates. Only read2, which corresponds to the 5’ end of CLIP tags, was used for analyses.

### Statistics

Experimental groups were compared using one-way ANOVA and Student’s T-tests. Differences were considered statistically significant at P<0.05.

## Supporting information

S1 Fig Testes from adult *Rbm46*^*FLAG/FLAG*^ mice were morphologically normal. (**A-B**) Similar to Bouin’s-fixed and PAS-stained testes from adult (P>60) WT (**A**) mice, those from *Rbm46*^*FLAG/FLAG*^ mice (**B**) contained normal complements of male germ cells (Spg = spermatogonium; Pl = preleptotene spermatocyte; L = leptotene spermatocyte; Z = zygotene spermatocyte; PS = pachytene spermatocyte; RS = round spermatid; ES = elongating spermatid; CS = condensing spermatid; SC = Sertoli cell nucleus) within that appropriate seminiferous tubule stages, indicated on each cross section in Roman numerals. Scale bar = 50 μm.
(TIF)

S2 Fig Schematic of Rbm46 whole-body and conditional KO alleles. (**A**) For whole-body KO allele, the deleted region is indicated by scissors. (**B**) For conditional KO allele, inserted loxP sites are represented by blue arrows. (**C**) RBM46 protein contains three RRMs, indicated in yellow.
(TIF)

S3 Fig Adult *Rbm46*^-/-^ ovaries lacked a germline. (A-B) PAS-stained ovaries from *Rbm46*^+/+^ and *Rbm46*^-/-^ mice, with genotypes indicated on each image. The cortex of an *Rbm46*^+/+^ ovary (**A**) contained numerous oocytes (white arrows) in follicles at various stages of development. In contrast, the *Rbm46*^+/+^ ovary lacked oocytes or organized follicles (**B**). Scale bar = 200 μm.
(TIF)

S4 Fig Adult *Rbm46*^-/-^ testes contained abundant Sertoli cells but lacked SYCP3+ meiotic spermatocytes. (**A-B**) GATA4+ Sertoli cells (green) were present in both *Rbm46*^+/-^ and *Rbm46*^-/-^ testes, but there were few TRA98+ (red) germ cells in *Rbm46*^-/-^ testes. (**C-D**) In contrast to *Rbm46*^+/-^ testes, there were no SYCP3+ (green) spermatocytes in *Rbm46*^-/-^ testes. (**E-F**) Using *Rbm46*^+/-^ and *Rbm46*^-/-^ testes, the numbers of germ cells (**E**) and % cell fate (**F**) were quantified. Nuclei were stained with DAPI (blue). Scale bar = 50 μm.
(TIF)

S5 Fig Conditional deletion of *Rbm46* with *Stra8*-Cre resulted in an adult spermatogenesis phenotype resembling that of whole-body KO mice. (**A-C**) Compared to controls, adult conditional KO testes were dramatically reduced in size. (**D**) Seminiferous epithelia from control mice (left panel) contained Sertoli cells as well as all advanced germ cell types, with examples marked including leptotene (Lep) and pachytene (Pac) spermatocytes as well as elongated spermatids (ES). In stark contrast, seminiferous epithelia of conditional KO testes contained only somatic Sertoli cells and a few spermatogonia (Spg). Scale bar = 50 μm.
(TIF)

S6 Fig Similar numbers of spermatogonia present in *Rbm46*^*+/-*^ and *Rbm46*^*-/-*^ testes at P6. (TIF)

S7 Fig Differential splicing events in *Rbm46*^*-/-*^ testes at P8. (**A**) Heatmap depicts 36 splicing events with a change in percent spliced in (PSI) of at least 15% in *Rbm46*^*-/-*^ relative to *Rbm46*^*+/+*^ testes at P8. (**B**) Distribution of the types of altered splicing events in *Rbm46*^*-/-*^ testes. Absolute number of changing events for each type shown on the chart. ALE = Alternative Last Exon; AFE = Alternative First Exon; Alt 3 = Alternative 3’ splice site; Alt 5 = Alternative 5’ splice site.
(TIF)

S8 Fig CLIP of *Rbm46*^FLAG/FLAG^ in P21 mouse testes. (**A**) SDS-PAGE of crosslinked immunoprecipitants and input from *Rbm46*^FLAG/FLAG^ and *Rbm46*^WT/WT^ testes. RNAs in the immunoprecipitants were ligated (on beads) with an RNA linker containing the IRDye 800CW fluorochrome to enable RNA visualization. (**B**) Corresponding anti-FLAG western blot of crosslinked immunoprecipitants and input from *Rbm46*^FLAG/FLAG^ and *Rbm46*^WT/WT^ testes following FLAG immunoprecipitation.
(TIF)

S1 Table List of differentially expressed genes in *Rbm46*^*-/-*^ testes at P8. (XLS)

S2 Table List of differential splicing events in *Rbm46*^*-/-*^ testes at P8. (XLS)

S3 Table List of genes bound by RBM46 at P21 whose mRNAs were differentially expressed in *Rbm46*^-/-^ testes at P8. (XLSX)
