# Supplementary figures and images for: The germ cell-specific RNA binding protein RBM46 is essential for spermatogonial differentiation in mice
Source: PLoS Genet. 2022 Sep 21;18(9):e1010416. doi: 10.1371/journal.pgen.1010416 (PMC9529142; doi:10.1371/journal.pgen.1010416)

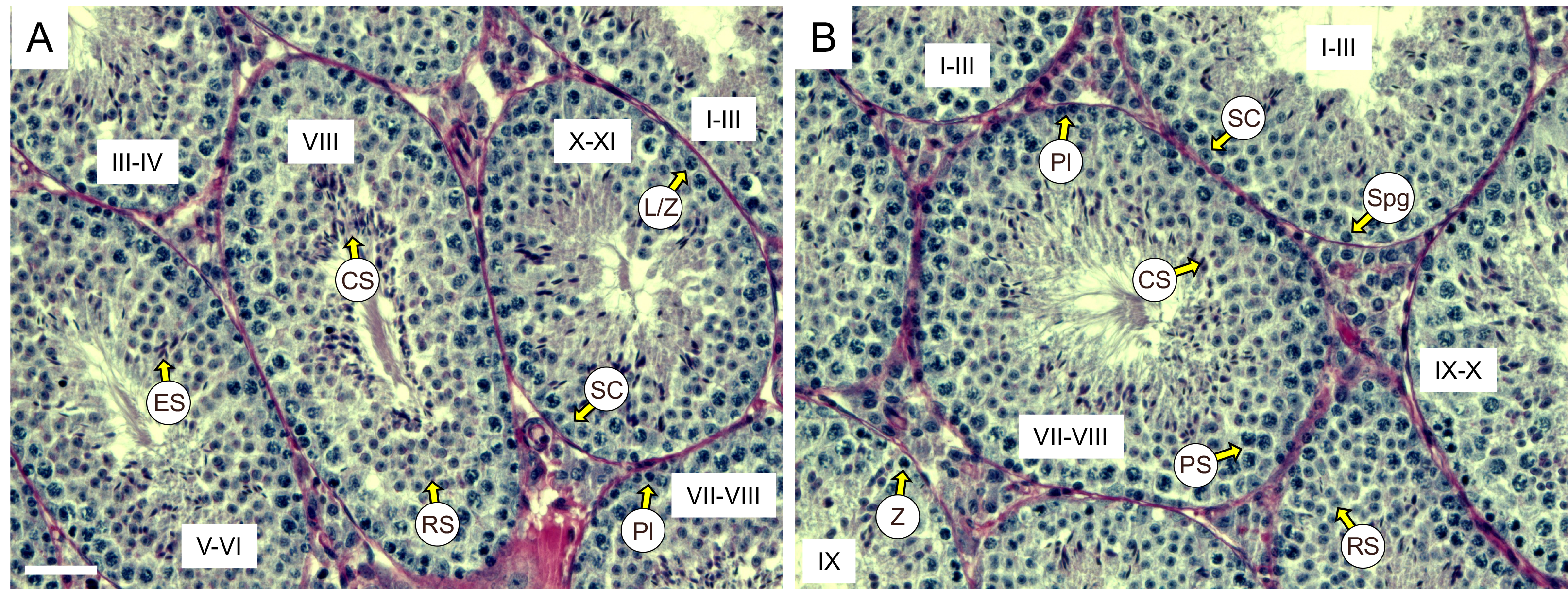

Supplement: S1 Fig — (A-B) Similar to Bouin’s-fixed and PAS-stained testes from adult (P>60) WT (A) mice, those from Rbm46FLAG/FLAG mice (B) contained normal complements of male germ cells (Spg = spermatogonium; Pl = preleptotene spermatocyte; L = leptotene spermatocyte; Z = zygotene spermatocyte; PS = pachytene spermatocyte; RS = round spermatid; ES = elongating spermatid; CS = condensing spermatid; SC = Sertoli cell nucleus) within that appropriate seminiferous tubule stages, indicated on each cross section in Roman numerals. Scale bar = 50 μm. (TIF) [file pgen.1010416.s001.tif]

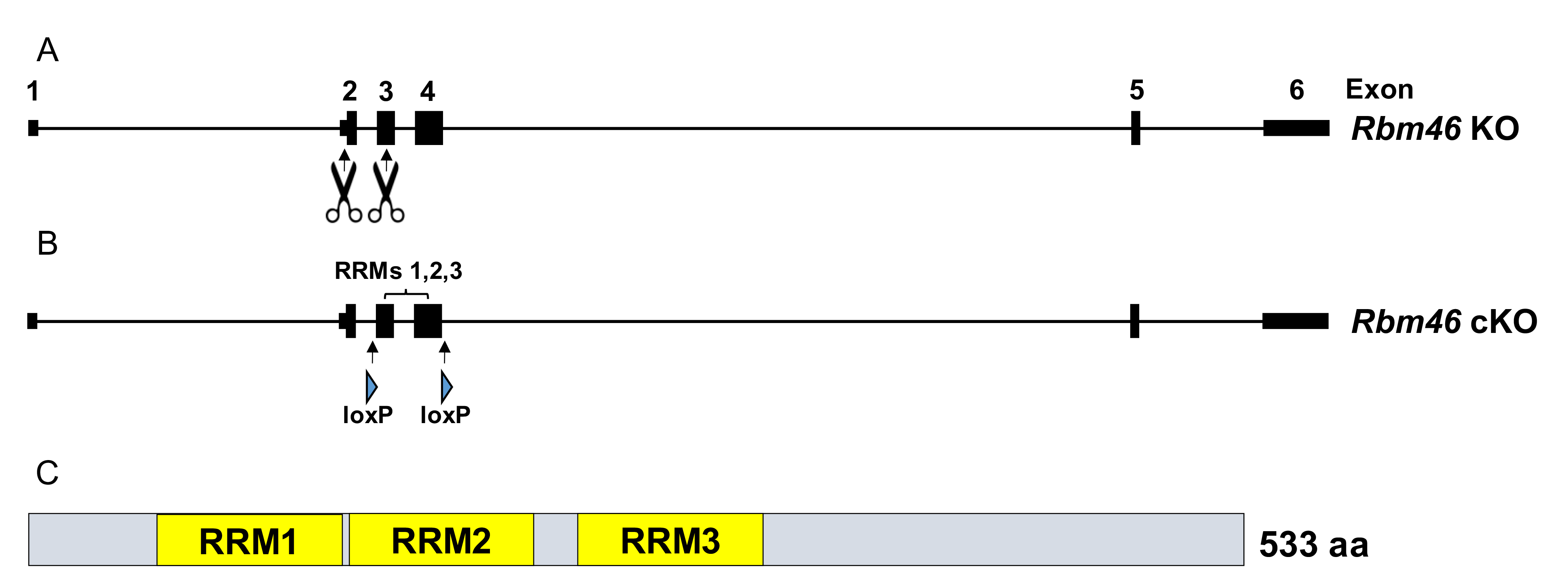

Supplement: S2 Fig — (A) For whole-body KO allele, the deleted region is indicated by scissors. (B) For conditional KO allele, inserted loxP sites are represented by blue arrows. (C) RBM46 protein contains three RRMs, indicated in yellow. (TIF) [file pgen.1010416.s002.tif]

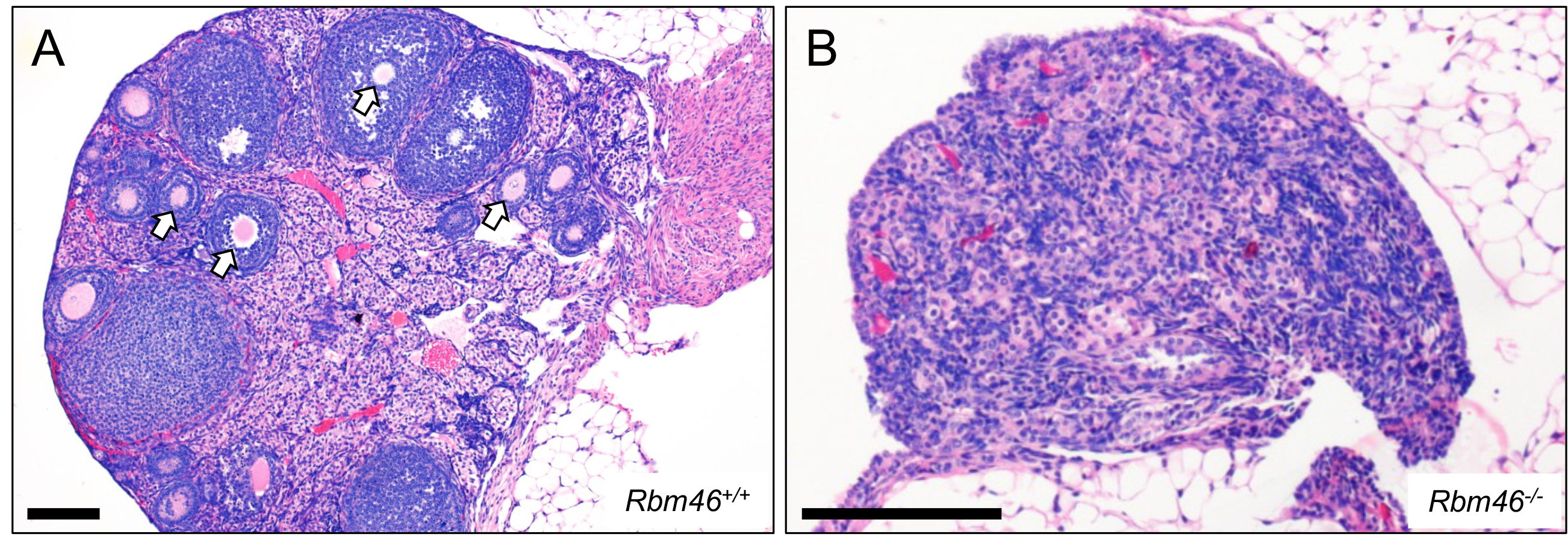

Supplement: S3 Fig — (A-B) PAS-stained ovaries from Rbm46+/+ and Rbm46-/- mice, with genotypes indicated on each image. The cortex of an Rbm46+/+ ovary (A) contained numerous oocytes (white arrows) in follicles at various stages of development. In contrast, the Rbm46+/+ ovary lacked oocytes or organized follicles (B). Scale bar = 200 μm. (TIF) [file pgen.1010416.s003.tif]

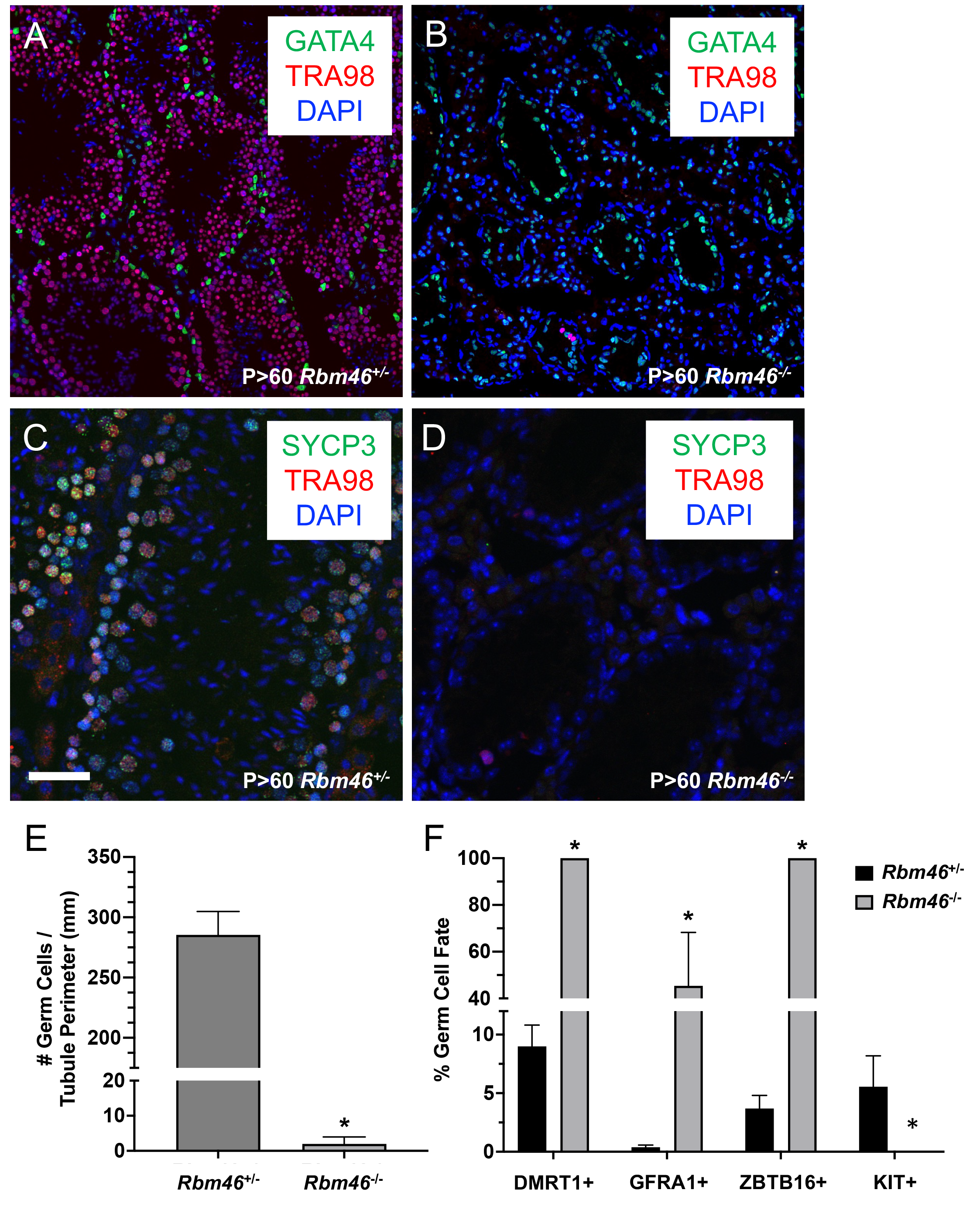

Supplement: S4 Fig — (A-B) GATA4+ Sertoli cells (green) were present in both Rbm46+/- and Rbm46-/- testes, but there were few TRA98+ (red) germ cells in Rbm46-/- testes. (C-D) In contrast to Rbm46+/- testes, there were no SYCP3+ (green) spermatocytes in Rbm46-/- testes. (E-F) Using Rbm46+/- and Rbm46-/- testes, the numbers of germ cells (E) and % cell fate (F) were quantified. Nuclei were stained with DAPI (blue). Scale bar = 50 μm. (TIF) [file pgen.1010416.s004.tif]

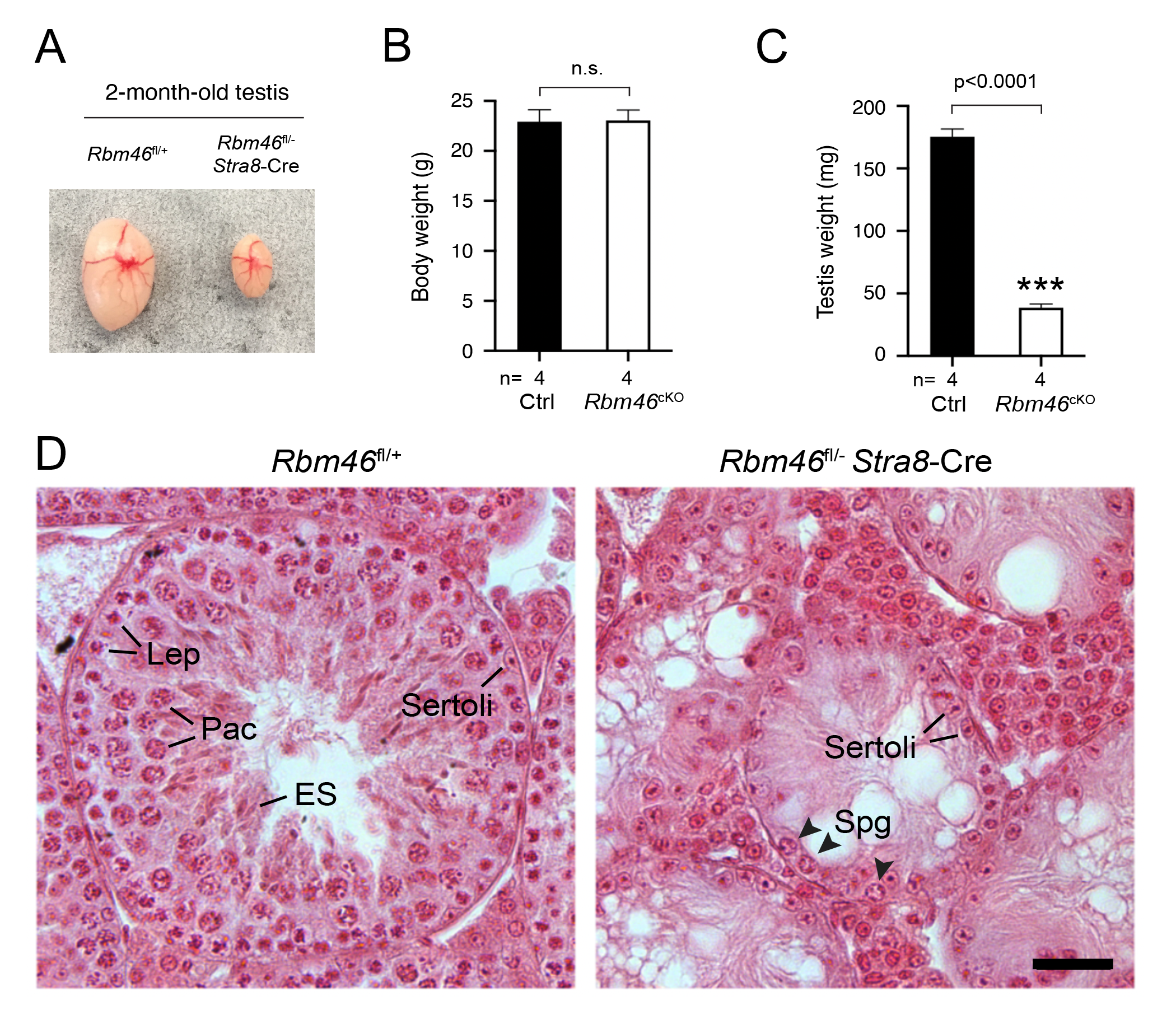

Supplement: S5 Fig — (A-C) Compared to controls, adult conditional KO testes were dramatically reduced in size. (D) Seminiferous epithelia from control mice (left panel) contained Sertoli cells as well as all advanced germ cell types, with examples marked including leptotene (Lep) and pachytene (Pac) spermatocytes as well as elongated spermatids (ES). In stark contrast, seminiferous epithelia of conditional KO testes contained only somatic Sertoli cells and a few spermatogonia (Spg). Scale bar = 50 μm. (TIF) [file pgen.1010416.s005.tif]

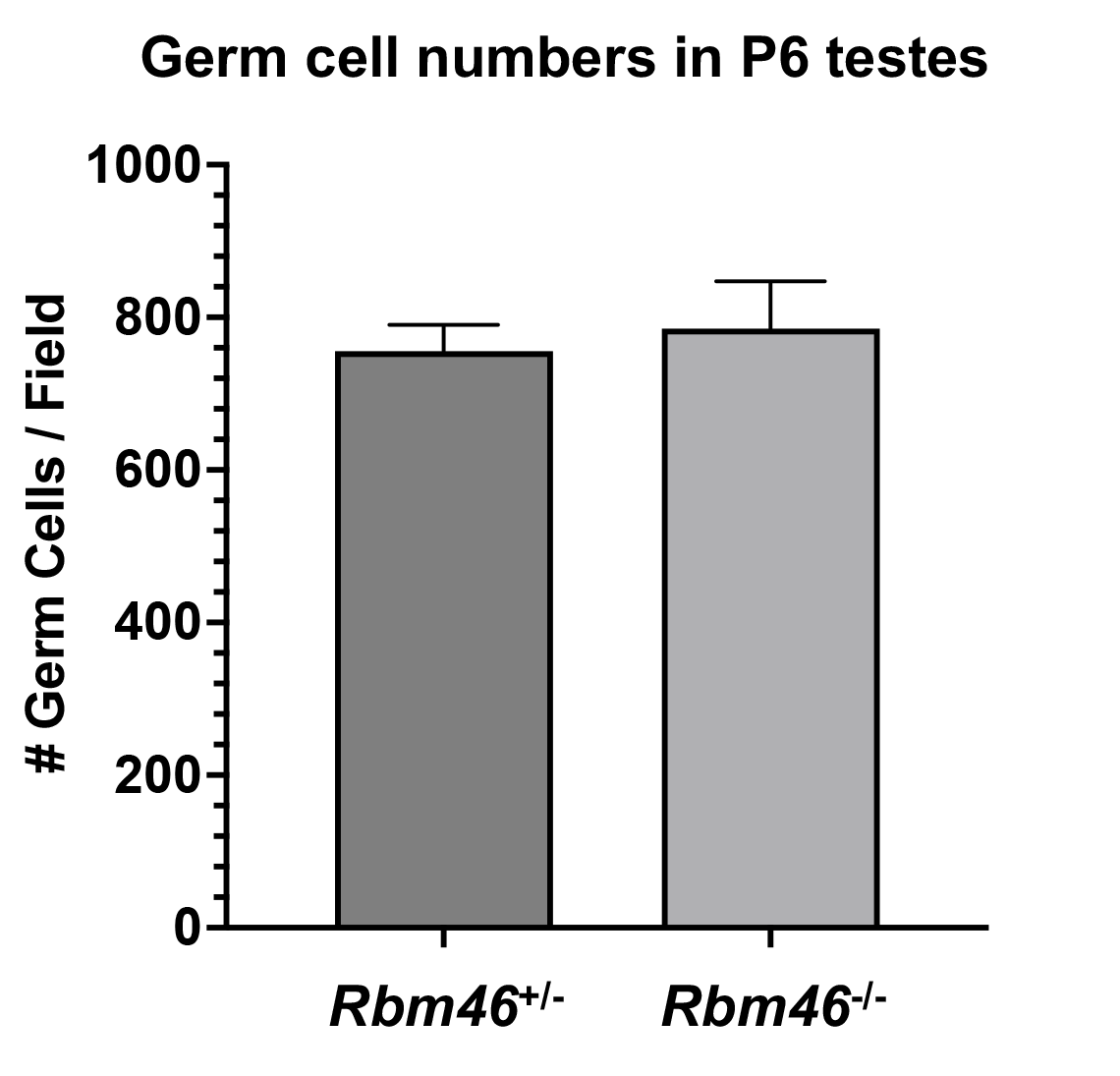

Supplement: S6 Fig — (TIF) [file pgen.1010416.s006.tif]

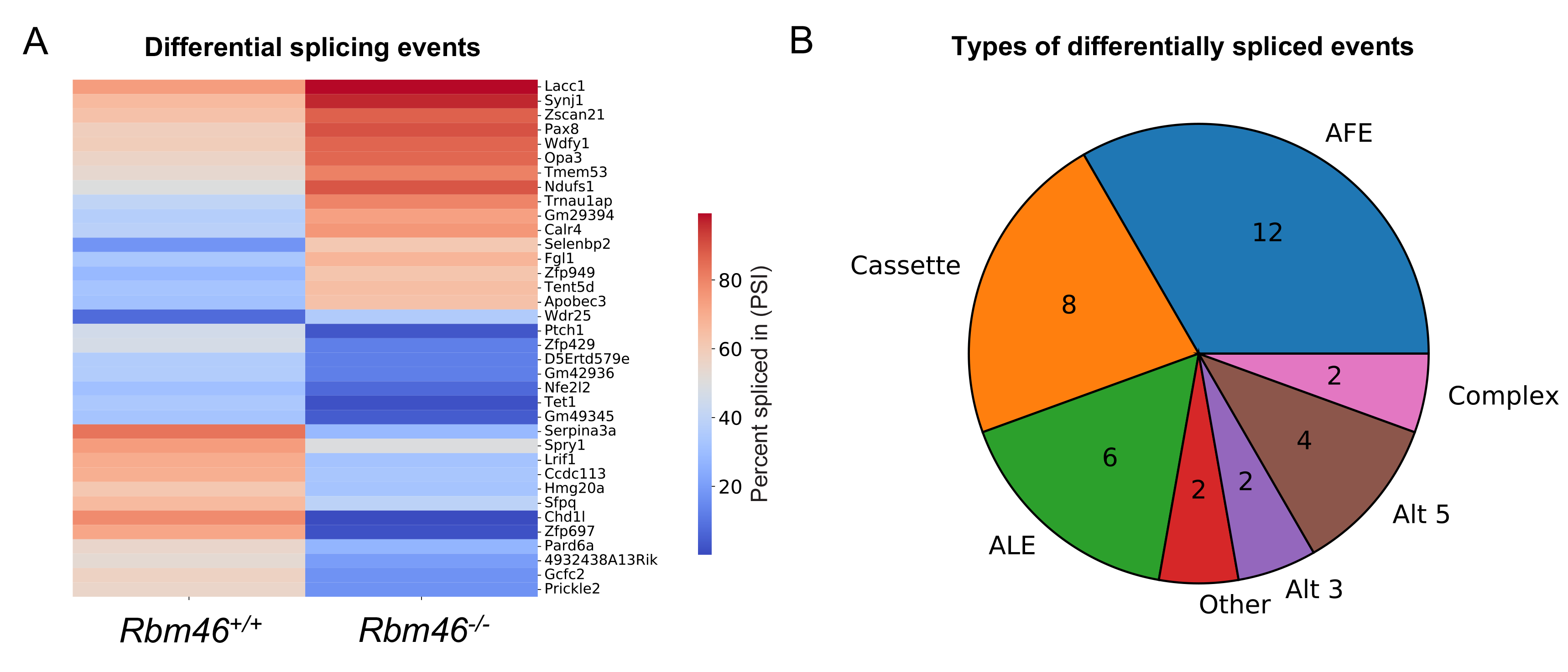

Supplement: S7 Fig — (A) Heatmap depicts 36 splicing events with a change in percent spliced in (PSI) of at least 15% in Rbm46-/- relative to Rbm46+/+ testes at P8. (B) Distribution of the types of altered splicing events in Rbm46-/- testes. Absolute number of changing events for each type shown on the chart. ALE = Alternative Last Exon; AFE = Alternative First Exon; Alt 3 = Alternative 3’ splice site; Alt 5 = Alternative 5’ splice site. (TIF) [file pgen.1010416.s007.tif]

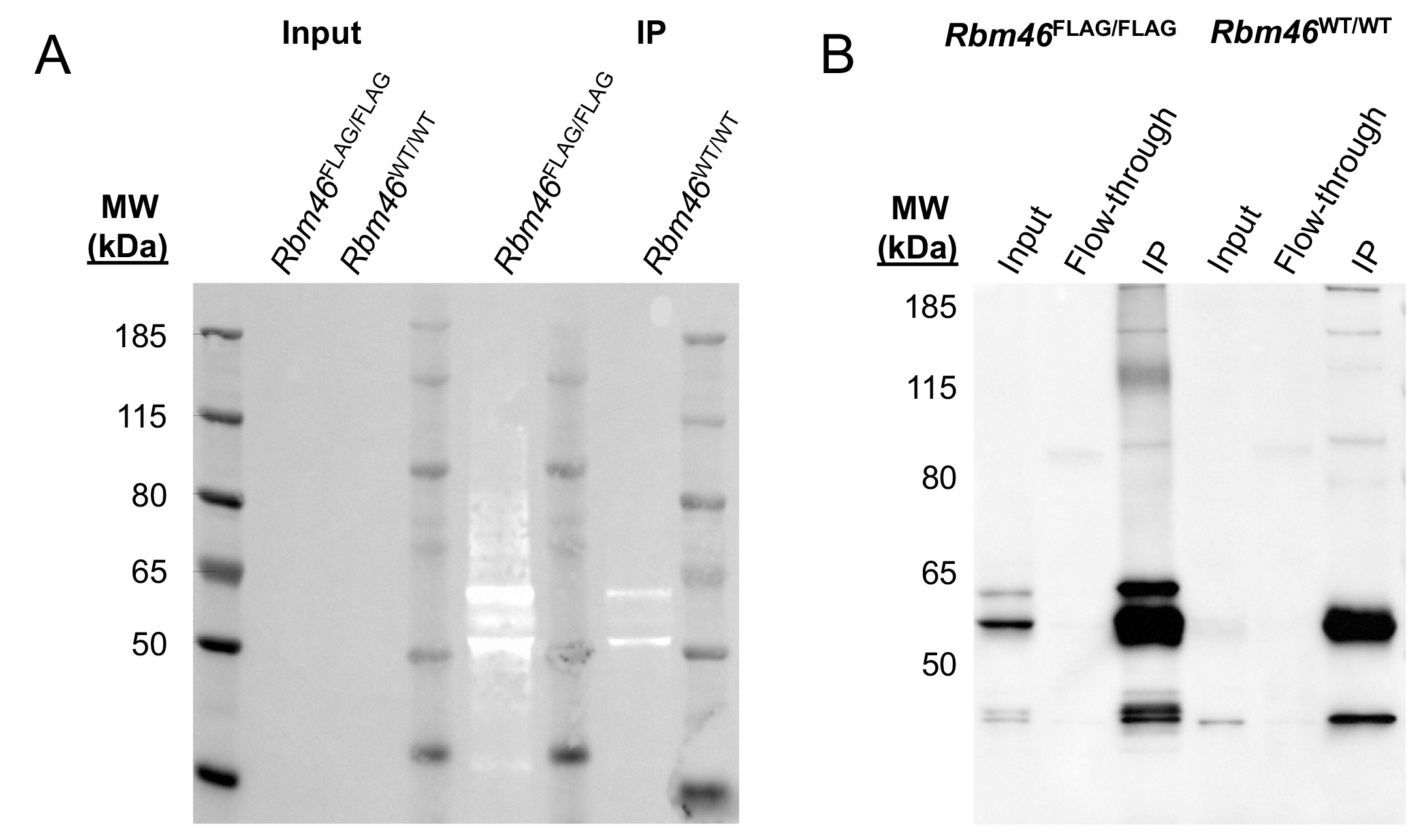

Supplement: S8 Fig — (A) SDS-PAGE of crosslinked immunoprecipitants and input from Rbm46FLAG/FLAG and Rbm46WT/WT testes. RNAs in the immunoprecipitants were ligated (on beads) with an RNA linker containing the IRDye 800CW fluorochrome to enable RNA visualization. (B) Corresponding anti-FLAG western blot of crosslinked immunoprecipitants and input from Rbm46FLAG/FLAG and Rbm46WT/WT testes following FLAG immunoprecipitation. (TIF) [file pgen.1010416.s008.tif]
